# Does planning help for execution? The complex relationship between planning and execution

**DOI:** 10.1371/journal.pone.0237568

**Published:** 2020-08-14

**Authors:** Zhaojun Li, Paul De Boeck, Jian Li

**Affiliations:** 1 Department of Psychology, The Ohio State University, Columbus, Ohio, United States of America; 2 Faculty of Psychology, Beijing Normal University, Beijing, China; Duke University, UNITED STATES

## Abstract

Planning and execution are two important parts of the problem-solving process. Based on related research, it is expected that planning speed and execution speed are positively correlated because of underlying individual differences in general mental speed. While there could also be a direct negative dependency of execution time on planning time, given the hypothesis that an investment in planning contributes to more efficient execution. The positive correlation and negative dependency are not contradictory since the former is a relationship across individuals (at the latent variable level) and the latter is a relationship within individuals (at the manifest variable level) after controlling for across-individual relationships. With two linear mixed model analyses and a factor model analysis, these two different kinds of relationships were examined using dependency analysis. The results supported the above hypotheses. The correlation between the latent variables of planning and execution was found to be positive and the dependency of execution time on planning time was found to be negative in all analyses. Moreover, the negative dependency varied among items and to some extent among persons as well. In summary, this study provides a clearer picture of the relationship between planning and execution and suggests that analyses at different levels may reveal different relationships.

## Introduction

From daily routine to professional life, we encounter problems to be solved almost all the time and everywhere. Problem solving helps us not only to eradicate issues but also to achieve success. In psychological research, a problem is described as having three general states: an initial state (seeing the problem), a goal state (problem solved), and an action state in between, with steps the problem solver takes to transform the initial state into the goal state that are often not obvious [1]. Correspondingly, problem solving involves a sequence of operations to transform the initial state into the goal state [2]. Good problem solving requires both accurate planning (finding the sequence of operations) and efficient execution (putting the plan into practice). Specifically, planning involves the ability of searching for a promising solution from a problem space [3]. Execution requires (a) keeping the plan in mind long enough to guide the action, and (b) actually carrying out the prescribed behavior [4]. Investigating planning and execution, and the relationship between these two will provide a better understanding of the nature of problem solving.

Research on the problem-solving process suggests that the quality of problem solving relies on both planning and execution. A representative of early problem-solving models is Pólya’s [5] four-step model, which consists of (1) understanding the problem, (2) planning, (3) carrying out the plan, and (4) checking the result. Afterwards, Stein [6] proposed the IDEAL model in which problem solving was defined as a process including five steps: (1) identify the problem, (2) define and represent the problem, (3) explore possible strategies, (4) act on the strategies, and (5) look back and evaluate the effects of activities. Based on a synthesis of previous problem-solving models [6–8], Pretz, Naples, and Sternberg [9] stated that the problem-solving process was a cycle with the following stages: (1) recognize or identify the problem, (2) define and represent the problem mentally, (3) develop a solution strategy, (4) organize the knowledge about the problem, (5) allocate mental and physical resources for solving the problem, (6) monitor the progress toward the goal, and (7) evaluate the solution for accuracy. As we see, no matter what model is adopted, the problem-solving process always contains planning (described as “explore possible strategies” or “develop a solution strategy” in some models) and execution (described as “carry out the plan” or “act on the strategies” in some models).

Even though the two indispensable parts of problem solving, planning and execution, are closely connected, there is little empirical research on the relationship between them. Fortunately, some studies can be indirectly informative. Danthiir, Wilhelm, and Roberts [10] found that the scores of cognitive tasks employed in their experiment had a general speed factor, indicating that there was a general mental speed for cognitive activities. In theory, mental speed is defined as the ability of carrying out mental processes to solve a cognitive problem at variable rates or increments of time [11]. Planning is a well-known cognitive ability [12], and execution is also a cognitive ability to keep the plan in mind while one is acting. Therefore, we expect the corresponding latent variables of planning speed and execution speed to be positively correlated due to individual differences in general mental speed. In other words, if one has higher general mental speed compared with others, the individual is expected to have both higher planning speed and higher execution speed.

On the other hand, planning is defined as the process of searching for a solution as efficient as possible among many alternatives [3, 13]. Therefore, given a certain person and a certain problem, it is reasonable to assume that more time spent on planning for the problem contributes to more efficient strategies to solve the problem and allows the execution to be subsequently faster. Accordingly, we expect planning time to have a negative effect on execution time after controlling for the positively correlated latent variables of planning and execution.

The combination of a positive relation and a negative relation between planning and execution is possible and not contradictory because the two relations concern different aspects of the data. Based on individual differences in general mental speed, individual problem solvers who are fast (or slow) on planning may also be fast (or slow) on execution. This is a positive correlation between the latent variables of planning speed and execution speed to be found across individuals. To examine such relations between constructs based on their latent variables is usually a research interest in the domain of measurement. However, despite the tendency to concentrate on the latent variable level, it is possible that apart from the association between the latent variables, there may also be a direct negative dependency of execution time on planning time (i.e., more planning time may facilitate execution) within the same problem-solving task for a given person.

A number of studies with dependency analysis provide a potential approach to test the above assumption [14–17]. In a dependency analysis, instead of focusing only on the relations at the latent variable level and assuming no residual dependency at the manifest variable level, researchers also estimate remaining relations among manifest variables which are called conditional dependency (i.e., the dependency between manifest variables conditional on the relations between latent variables). It has been shown in those studies that the relations between manifest variables may not always be fully explained by latent variables, and there may exist additional dependency information in the data which is not captured by the latent variables. Dependency analysis can be used for a simultaneous investigation of the relations between planning and execution at the latent variable level and at the manifest variable level. Our hypothesis is that the two types of relations have opposite signs: a positive correlation between the latent variables of planning speed and execution speed and a negative conditional dependency of execution time on planning time.

The aim of this study is to use dependency analysis to fill the gaps in the literature regarding the relationship between the two essential components of problem-solving: planning and execution. We recorded the respective times spent on planning and execution during the problem-solving process in a game-based assessment that allows us to separate these two components. In this way, the relationship between planning and execution can be investigated.

## Method

### Measures

**Fig 1:**
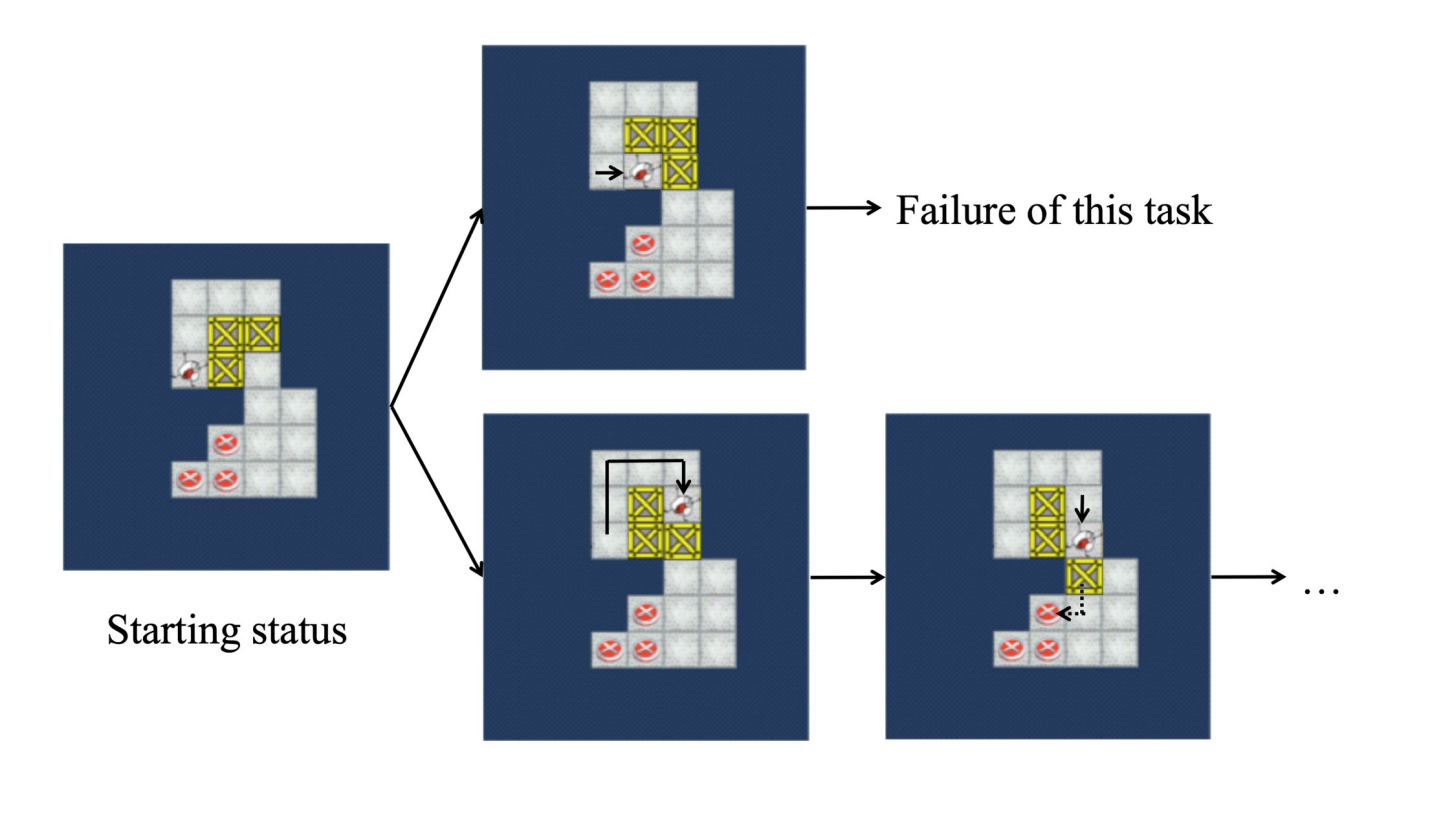
An example of the Sokoban task.

A game-based assessment tool was adopted to measure planning time and execution time. This assessment tool was developed by Li, Zhang, Du, Zhu, and Li [13] from a Japanese puzzle game—Sokoban. There are 10 tasks in the assessment. A task is shown in Fig 1 as an example. Every task of the Sokoban game consists of a pusher, a small set of boxes, and the same number of target locations. Players are instructed to manipulate the pusher to push all the boxes into the target locations. The pusher cannot push two or more boxes at the same time. Pulling boxes is not allowed.

In the assessment, the first move in every task was redesigned to be a crucial move, so that there is only one correct first move and any other move results in failure. For example, in Fig 1, if the pusher moves the most nearby box toward the right first, the player will encounter an impasse (note that it is not allowed to push two or more boxes or to pull boxes). The only correct first move is to push the top-right box downwards. In the instructions, participants were told that their move could not be taken back and that they were advised to plan before the first move to avoid an impasse. The time from the beginning of a task to the first move was recorded as planning time, and the time from the first move to the completion of each task was recorded as execution time. There was no time limit for these tasks.

### Participants

The participants were 266 college students (65 males, 201 females) from a Chinese university. Their ages ranged between 18 and 31 (mean = 20.70, *SD* = 1.56). The pass rates of the 10 tasks ranged from 80% to 96% per task. Out of the 266 participants, 11 passed five or fewer tasks, 11 passed six tasks, 15 passed seven tasks, 40 passed eight tasks, and 70 passed nine tasks.

Only the data for the 119 participants who completed all 10 tasks successfully will be focused on in the current study (43 males, 76 females). Their ages ranged between 18 and 27 (mean = 20.70, *SD* = 1.48). The reason for focusing on successful trials is that there was no execution time available in the case of failure because one was stuck after an incorrect move.

To check the effect of the reduced sample size (from 266 to 119) due to non-completion of tasks, we also conducted all the analyses in this study for participants who completed fewer tasks: either at least the same nine (N = 133), the same eight (N = 142), the same seven (N = 153), the same six (N = 165), or the same five tasks (N = 177). The results were consistent with those for the 119 participants who completed all tasks. Therefore, the results shown in this study can be generalized to the larger set of participants who did not complete all tasks.

### Ethics statement

This study was approved by the ethics board of Faculty of Psychology in Beijing Normal University and was in accordance with the ethical principles and guidelines recommended by the American Psychological Association. The written forms of consent were obtained from all individual participants included in the study and the data were analyzed anonymously.

### Methods of analysis

Before analyzing the data, a Kolmogorov-Smirnov normality test was first conducted [18]. For planning time, *D* = 0.21, *p* < 2.20*10^−16^, and for execution time, *D* = 0.17, *p* <2.20*10^−16^, indicating that the distributions of both planning time and execution time were not normal.

The Box-Cox power transformation is commonly used to provide a statistically optimal data transformation (e.g., log and inverse), which normalizes the data distribution [19]. Therefore, this method was applied, and the result showed that a logarithmic transformation was appropriate. The logarithmic transformation is one of the most common ways to make data more consistent with the statistical assumptions in psychometrics [20].

**Fig 2:**
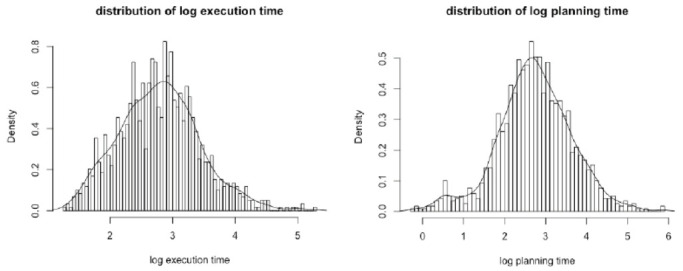
Distributions of logarithm of execution time and logarithm of planning time.

After logarithmic transformation, for execution time, *D* = 0.03, *p* = 0.07; the null hypothesis of a normally distributed log execution time was not rejected at the significance level of 0.05. For planning time, *D* = 0.04, *p* = 0.0004; even though the normality hypothesis was still rejected at the significance level of 0.05, the violation of normality was largely alleviated compared with the original data. Besides, the resulting distributions were very similar to the normal distribution (see Fig 2). Thus, log-transformed data were used in the following analyses.

In line with a recently proposed multiverse strategy [21], more than one approach were used for the data analyses. In this way, we could increase the research transparency and verify the robustness of our findings. We chose to analyze the data with two different linear mixed model (LMM) analyses (Analyses 1 and 2) and with a factor model analysis (Analysis 3). The data of this study and the code for all three analyses are available on https://osf.io/8pw3d/.

#### Analysis 1: LMM with observed planning time as a covariate

We adopted a LMM approach to explore the relationship between planning and execution. Several models were estimated. The first model is a LMM with correlated random intercepts of planning and execution in which the correlation is estimated across both persons and items (i.e., tasks). Note that the correlated random intercepts across persons are equivalent to correlated latent variables of planning and execution in a factor model. The hypothesis that planning and execution are positively correlated across individuals can be tested through the correlation between the random person intercepts. The second model is again a LMM with correlated random intercepts but also includes a direct effect of planning time on execution time for each pair of persons and tasks. In this way, we can investigate whether there is conditional dependency of execution time on planning time that cannot be captured by the relation between the random intercepts.

The first model, Model 1, contains a planning component and an execution component. Both components have a random person intercept and a random item intercept, as shown in Eqs 1A and 1B, with covariance matrices defined after the equations are presented:

$$E(\log\;{PT}_{pi})=\mu (p)+{\tau (p)}_{p}+{\beta (p)}_{i},$$ (1A)

$$E(\log\;{ET}_{pi})=\mu (e)+{\tau (e)}_{p}+{\beta (e)}_{i},$$ (1B)

where E(logPT_pi_) is the expected value of the logarithm of the planning time for respondent *p* and item *i*, μ(p) is the fixed intercept for planning time, τ(p)_p_ is minus the specific planning speed (a random person intercept) of respondent *p*, β(p)_i_ is the time intensity for planning (a random item intercept) of item *i*; E(logET_pi_) is the expected value of the logarithm of the execution time for respondent *p* and item *i*, μ(e) is the fixed intercept for execution time, τ(e)_p_ is minus the specific execution speed (a random person intercept) of respondent *p*, and β(e)_i_ is the time intensity for execution (a random item intercept) of item *i*. The distributions of the random intercepts are bivariate normal, (τ(p)_p_, τ(e)_p_) ~ BVN(**0**, **Σ**_**τ**_), (β(p)_i_, β(e)_i_) ~ BVN(**0**, **Σ**_**β**_), allowing for a correlation between planning and execution time based on individual differences and item differences, respectively.

The second model, Model 2, differs from Model 1 in that it includes conditional dependency of execution time on planning time. The conditional dependency is a direct effect of planning time on execution time conditional on the random intercepts of planning and execution. To further inspect the property of the dependency, we proposed three variants of Model 2 with: either a global dependency constant across persons and items (Model 2a, with Eq 1C), or person-specific dependencies (Model 2b, with Eq 1D), or item-specific dependencies (Model 2c, with Eq 1E). In Model 2a, the dependency is a stable direct effect of planning on execution. Model 2b, however, assumes that the dependency of execution on planning may be stronger for some people than for others. Similarly, Model 2c implies that some items allow planning to contribute more to execution (i.e., stronger dependency) than other items do. The model equation for the planning time in Model 2 is the same as in Model 1, but for the execution time, the (logarithm of) observed planning time in the same item and of the same person is added as a predictor. For all three variants of Model 2, an overall fixed dependency parameter will be estimated, and for Models 2b and 2c, random deviations from the overall dependency are allowed for persons and items, respectively. In this way, Model 2a is nested in Models 2b and 2c, which makes model comparison easier.

As explained above, the equation for planning time (1a) is the same for all models (Model 1, Models 2a, 2b, and 2c), while for execution time in Model 2, one of the following three replaces Eq 1B of Model 1:

$$E(\log\;{ET}_{pi})=\mu (e)+{\tau (e)}_{p}+{\beta (e)}_{i}+\omega \;\log\;{PT}_{pi},$$ (1C)

$$E(\log\;{ET}_{pi})=\mu (e)+{\tau (e)}_{p}+{\beta (e)}_{i}+\omega \;\log\;{PT}_{pi}+\omega_{p}\;\log\;{PT}_{pi},$$ (1D)

$$E(\log\;{ET}_{pi})=\mu (e)+{\tau (e)}_{p}+{\beta (e)}_{i}+\omega \;\log\;{PT}_{pi}+\omega_{i}\;\log\;{PT}_{pi},$$ (1E)

where ω is the overall fixed dependency of the logarithm of execution time on the logarithm of planning time, ω_p_ is a person-specific deviation from the overall dependency, and ω_i_ is an item-specific deviation from the overall dependency. The specific deviations ω_p_ and ω_i_ are random and normally distributed with a mean of zero and a variance of σ_ω(p)_^2^ and σ_ω(i)_^2^, respectively. In the same way as for Model 1, the distributions of the random intercepts are bivariate normal, (τ(p)_p_, τ(e)_p_) ~ BVN(**0**, **Σ**_**τ**_), (β(p)_i_, β(e)_i_) ~ BVN(**0**, **Σ**_**β**_).

The person-specific dependencies and the item-specific dependencies are modeled as independent of the random intercepts. However, we have also estimated models with correlations between the random dependencies and the random intercepts since the correlations between item-specific dependencies and item intercepts were examined in previous studies [15,16]. The likelihood ratio test found no significant difference between the models with and without the correlations between the item-specific dependencies and the random item intercepts of planning and execution. A possible reason for the non-significant result was that the correlations were based on only ten pairs of item-specific dependencies and random item intercepts. To investigate the correlations across items, we may need a larger number of items. For the correlations between the person-specific dependencies and the random person intercepts of planning and execution, we encountered estimation problems in the form of a degenerate solution. This degenerate solution was most likely due to a very small estimated variance of the person-specific dependencies, which led to unreliable correlations between the person-specific dependencies and the random person intercepts. To investigate the correlations across persons, a substantial variance of the person-specific dependencies may be needed. Given the above reasons, we decided to work with the models without the correlations between the random dependencies and the random intercepts.

**Fig 3:**
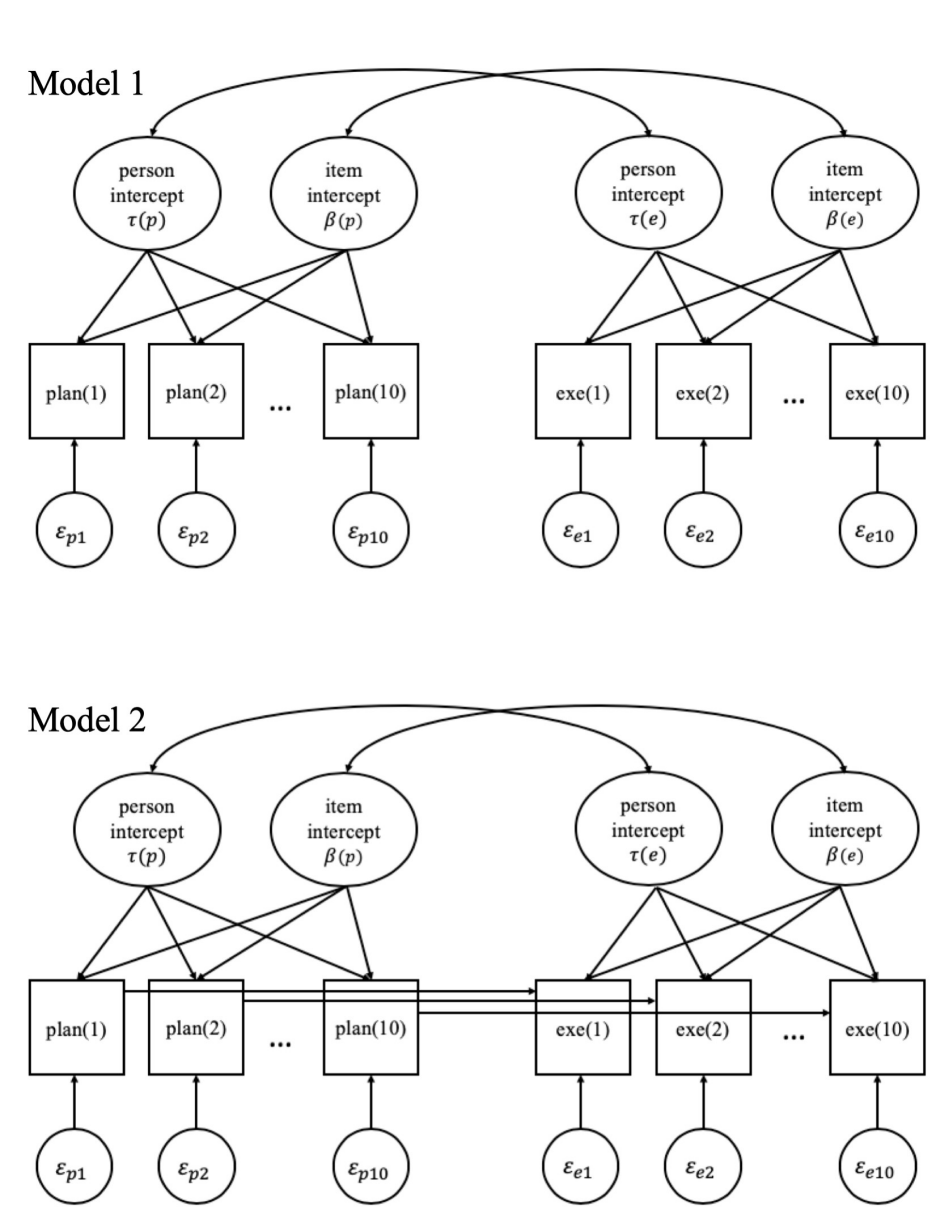
Path diagrams for the models in Analysis 1. Model 1 is the model without conditional dependency (the ND model). Model 2 has three variants of the direct effect arrows from observed planning time to observed execution time. Either the direct effect is constant (the GD model), or it varies across persons (the PSD model), or it varies across items (the ISD model).

To test for the presence of conditional dependency, Model 1, the no dependency model (ND model) as defined by Eqs 1A and 1B, was compared with the three variants of Model 2 (a, b, c), where (a) is the general dependency model (GD model) defined by Eqs 1A and 1C, (b) is the person-specific dependency model (PSD model) defined by Eqs 1A and 1D, and (c) is the item-specific dependency model (ISD model) defined by Eqs 1A and 1E. In addition, the GD model was compared with the PSD and ISD models to further explore possible person and item differences of the conditional dependency. Fig 3 gives a graphical presentation of the models without and with the conditional dependency of the observed execution time on the observed planning time. All these models were estimated with the lme4 package in R [22].

#### Analysis 2: LMM with residual planning time as a covariate

In the dependency models of Analysis 1, the observed planning time was used as a covariate to predict the observed execution time, while in Analysis 2, the residual planning time (the concept of residual planning time will be explained later) replaced the observed planning time based on the following reasoning. In all models thus far (Models 1 and 2), the observed planning time consists of three random components: a random person intercept (representing the planning latent variable), a random item intercept (representing the item time intensity for planning), and an error term (estimated as the residual). The random person intercept and the random item intercept take care of planning time differences across persons and across items, which are the main effects of persons and items. The residual planning time is the difference between the observed planning time and the expected planning time given respondent *p* and item *i* and based on Eq 1A. The residual reflects variation that is neither due to a person’s average planning time nor to an item’s average time intensity of planning. Instead, the residual reflects extra variation across pairs of respondent *p* and item *i*. In other words, the residual planning time is the planning time corrected for individual differences and item differences. The effect of the residual planning time on the execution time demonstrates whether some extra planning pays off to allow faster execution, independent of the values of the random intercepts. Therefore, in Analysis 2, we focused exclusively on the residual planning time as a predictor for the execution time. The dependency is the effect of the residual planning time on the execution time. By correcting for the random intercepts (i.e., individual differences and item differences), the residual planning time is supposed to have an effect on the execution time purely at the manifest variable level.

Different from Analysis 1, we worked with two steps here. First, the residual planning times were determined based on a model for only the planning time, following Eq 1A. Next, in the second step, we focused on models for only the execution time, using the residual planning time from the first step as a predictor. The model for planning time is the same as in Models 1 and 2 and is formulated in Eq 1A. Based on that model, the residual planning time (called *RES* in the equations) was calculated as follows:

$${RES}_{pi}=\log\;{PT}_{pi}-E(\log\;{PT}_{pi}),$$ (2A)

where logPT_pi_ is the logarithm of the observed planning time for respondent *p* and item *i*, and E(logPT_pi_) is the expected value of log *PT_pi_* obtained from the model as defined in Eq 1A and based on the maximum a posteriori method (as used in the ranef function in lme4 R package [22]). After obtaining the residual planning time, the models for execution time were formulated as follows:

No dependency (ND):

$$E(\log\;{ET}_{pi})=\mu (e)+{\tau (e)}_{p}+{\beta (e)}_{i},$$ (2B)

General dependency (GD):

$$E(\log\;{ET}_{pi})=\mu (e)+{\tau (e)}_{p}+{\beta (e)}_{i}+\omega {RES}_{pi},$$ (2C)

Person-specific dependency (PSD):

$$E(\log\;{ET}_{pi})=\mu (e)+{\tau (e)}_{p}+{\beta (e)}_{i}+\omega {RES}_{pi}+\omega_{p}{RES}_{pi},$$ (2D)

**Fig 4:**
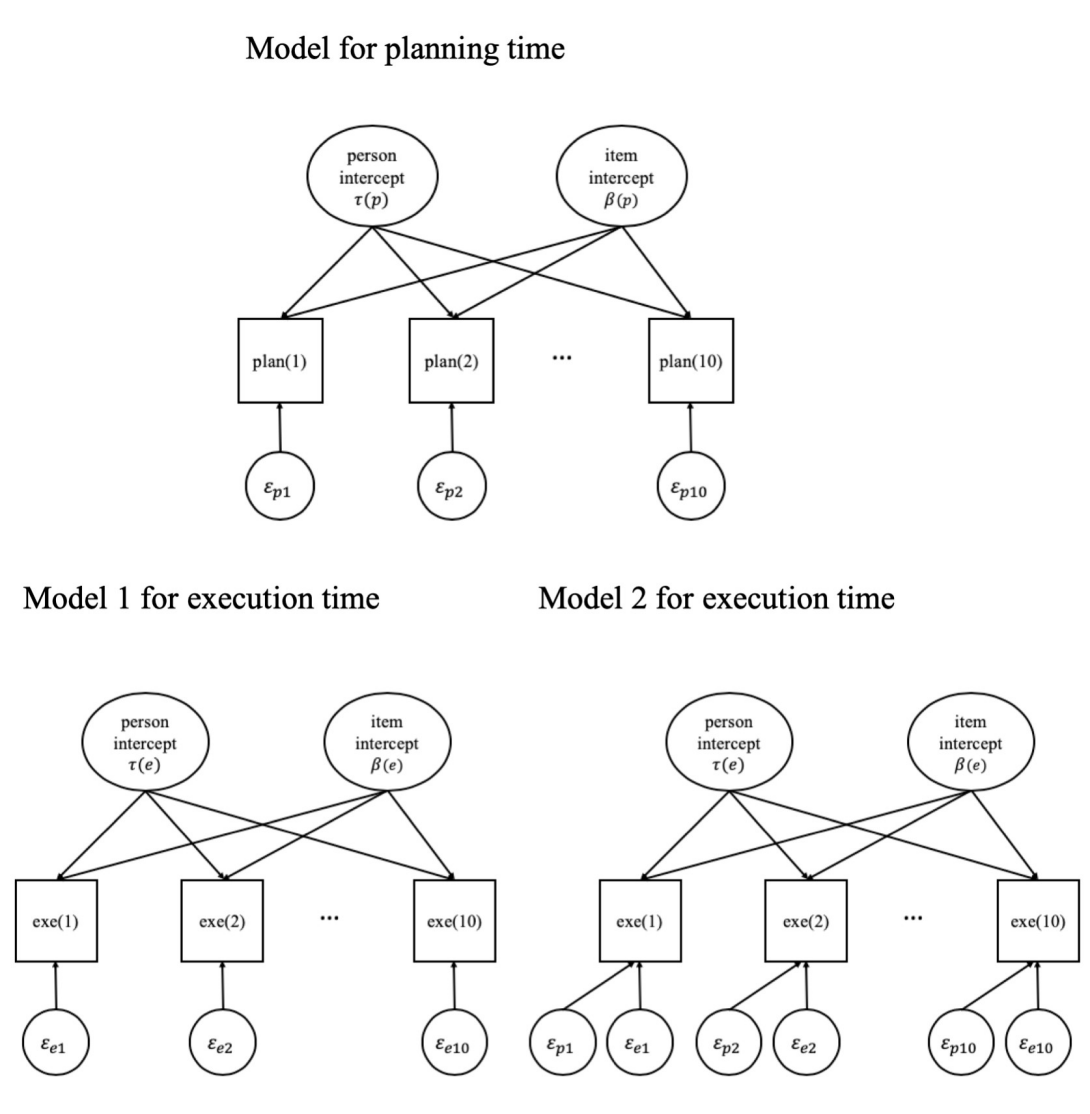
Path diagrams for the models in Analysis 2. The model for planning time is shown on top. Model 1 for execution time is the model without conditional dependency (the ND model). For Model 2 there are three variants: either the effect of the residual planning time is constant (the GD model), or it varies across persons (the PSD model), or it varies across items (the ISD model).

Item-specific dependency (ISD):

$$E(\log\;{ET}_{pi})=\mu (e)+{\tau (e)}_{p}+{\beta (e)}_{i}+\omega {RES}_{pi}+\omega_{i}{RES}_{pi},$$ (2E)

where the meaning of the notations is the same as in Eqs 1B to 1E and Eq 2A. However, unlike for the previous models, there are no bivariate distributions for planning and execution because in the second step of the analysis, only the execution time is modeled. Instead, we have τ(e)_p_ ~ N(0, σ_τ_^2^) and β(e)_i_ ~ N(0, σ_β_^2^). Based on the same reasoning as for Analysis 1, we have not estimated the correlations of the person-specific dependencies and the random person intercepts. Also, in a similar way as for Analysis 1, the model allowing for correlations between the item-specific dependencies and the random item intercepts was not supported by a likelihood ratio test as it was not significantly better than the model without the correlations. The models without and with dependencies of the observed execution time on the residual planning time are graphically presented in Fig 4. All models for the two steps were estimated with the lme4 package in R [22].

#### Analysis 3: Factor model analysis of the relationship between planning and execution

**Fig 5:**
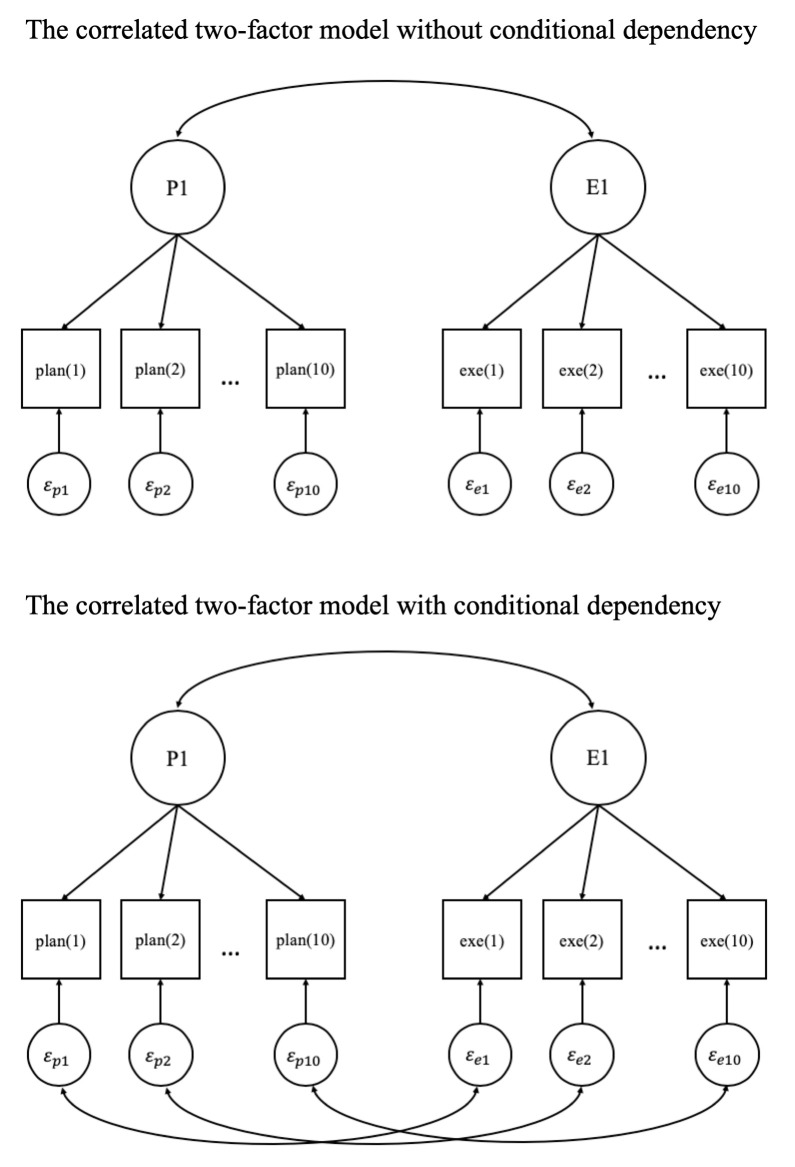
Factor models of the relationship between planning and execution.

In this analysis, the relationship between planning and execution was investigated with two factor models for the logarithm of planning time and the logarithm of execution time (see Fig 5). The first model is a *correlated two-factor model* in which all ten planning times load on one factor (factor P1) and all ten execution times load on the other factor (factor E1). In the second model, the *residual correlation* between observed planning time and observed execution time (i.e., between the log of these times) is added per item. The factor models were estimated with the R package lavaan that is extensively used for confirmatory factor analysis [23].

The residual correlations in the second factor model and the item-specific dependencies in previous analyses are different ways to capture the item-wise variation of the conditional dependency. Therefore, we correlated the estimated residual correlations from the second factor model with the item-specific dependencies from the ISD model in Analyses 1 and 2 to investigate whether they could correspond. High correlations between dependencies as estimated from different models would be an indication that the item-wise dependencies are a robust result and not artifacts from the analysis approach.

## Results

### Results of Analysis 1

The descriptive statistics are shown in Table 1. There is substantial variation of the planning time and execution time across participants. The modeling results are as follows. In Model 1 and in the three versions of Model 2, positive correlations were found between random person intercepts of planning and execution, indicating that the latent variables of planning and execution are positively correlated (Table 2). In other words, participants who use more time to plan will also use more time to execute compared to others (note that this is a correlation based on overall inter-individual differences), which is consistent with the hypothesis regarding general mental speed for planning and execution.

**Table 1:** Planning time and execution time for each item (sec).

	Planning time		Execution time			Planning time		Execution time	
	M	SD	M	SD		M	SD	M	SD
Item1	23.02	26.89	31.17	23.42	Item6	22.78	34.88	13.26	8.79
Item2	21.70	18.50	9.15	7.70	Item7	25.38	23.43	22.40	11.33
Item3	15.41	11.34	12.21	11.28	Item8	21.10	27.64	22.51	17.26
Item4	19.36	18.22	13.76	9.86	Item9	24.76	25.96	30.30	22.00
Item5	19.88	23.13	19.14	9.36	Item10	35.58	42.25	29.13	20.53

**Table 2:** Correlations and dependencies of planning and execution (Analysis 1).

		ND model	GD model	PSD model	ISD model
Correlation	across persons	0.35	0.51	0.51	0.51
	across items	0.36	0.40	0.40	0.53
Fixed dependency	estimate	-	-0.09	-0.09	-0.09
	SE	-	0.02	0.02	0.04
	t value	-	-4.06	-4.04	-2.49
Random dependency	SD	-	-	0.02	0.09

For all models, positive correlations between random item intercepts of planning and execution have also been found, which means that planning and execution are positively correlated across items. It is reasonable to assume that participants would spend more (or less) time on both planning and execution if they deal with an item with a longer (or shorter) route compared with other items, which brings about positive correlations between planning and execution across items. Accordingly, we would expect the route length of items to be positively correlated with both the logarithm of planning time and the logarithm of execution time. Following is a simple analysis to check this assumption. By using the average number of steps per item as the indicator of the route length of the item, we have found that the correlation between the route length and the logarithm of planning time is 0.59, and the correlation between the route length and the logarithm of execution time is 0.97. Furthermore, after adding the route length as a covariate into the ND, GD, PSD, and ISD models, the correlations of planning and execution across items were reduced to -0.37, -0.09, -0.09, and 0.10, respectively, which suggests that the positive correlation between planning and execution across items may stem from the route length.

Interestingly, when we focus on the conditional dependency, the fixed dependency parameter *ω*, which is also the mean of the person-specific and item-specific dependencies, is negative (see fixed dependency estimate in Table 2). The fixed dependency is the mean direct effect of planning on execution average over persons and items and is conditional on the random intercepts (i.e., independent of differences between persons and between items). The negative dependency implies that in general, spending more time on planning is associated with less time for the execution, after controlling for individual differences and item differences. As shown in Table 3, all three dependency models have better (i.e., smaller) goodness-of-fit indices (AIC and BIC) compared to the ND model without dependency. In line with the goodness-of-fit indices, the likelihood ratio test shows that the dependency models fit the data significantly better than the ND model does. It should be noticed that in the PSD and ISD models, the dependency is a random effect (either across persons or across items) and have a positive variance while the ND model implies no dependency and, thus, a zero dependency variance. This means that the ND model constrains the value of the dependency variance to the boundary of its parameter space, as the variance cannot be negative. According to Pinheiro and Bates [24] and Bates [25], in the likelihood ratio test, a bounded random effect variance violates the asymptotic chi-square reference distribution of the null hypothesis and makes the *p*-value “conservative”. In other words, the *p*-value is larger than it is supposed to be. In our model comparison, the likelihood ratio test shows significant differences between the dependency models and the ND model, even with conservative *p*-values when the PSD and ISD models are involved. Therefore, the hypothesis that the execution time has a negative conditional dependency on the planning time is supported by the results.

**Table 3:** Goodness of fit of the ND, GD, PSD, and ISD models (Analysis 1).

Model	AIC	BIC	Log-likelihood	Likelihood ratio test	
				Compared to ND	Compared to GD
ND	4221.79	4273.77	-2101.90	-	-
GD	4211.35	4269.10	-2095.67	X^2^(1) = 12.45***	-
PSD	4213.31	4276.84	-2095.66	X^2^(2) = 12.48**	X^2^(1) = 0.03
ISD	4203.02	4266.54	-2090.51	X^2^(2) = 22.78***	X^2^(1) = 10.33**

*p<0.05

**p<0.01

***p<0.001.

The estimated standard deviations of the random dependencies as shown in Table 2 reflect that compared with the item-specific dependency, the variation of the person-specific dependency is very small. Whether the conditional dependency varies across persons and items can be formally tested by comparing the PSD and ISD models with the GD model based on goodness-of-fit indices and the likelihood ratio test. As Table 3 shows, the ISD model has both smaller AIC and smaller BIC compared to the GD model. Besides, the likelihood ratio test indicates that the ISD model fits the data significantly better than the GD model even with a conservative *p*-value due to the bounded dependency variance. Accordingly, the model comparison supports that the dependency varies across items. The result is different for the test of the person-specific dependency. The AIC and BIC of the PSD model are slightly worse than those of the GD model. The likelihood ratio test shows no significant difference between the PSD model and the GD model. However, caution should be exercised here as the test is conservative because of the bounded dependency variance. Bates [25 p. 44] stated that “in the worst-case scenario the chi-square-based *p*-value will be twice as large as it should be”. The *p*-value of the likelihood ratio test for the PSD and GD models is 0.14, and even in the worst-case scenario, an effective *p*-value of 0.07 would still be larger than the significance level 0.05. Therefore, the null hypothesis of fixed dependency across persons cannot be rejected. Based on this result and the goodness-of-fit indices, there is no support for the person-specific dependency.

In addition, a supplementary analysis has been conducted to examine whether the covariates, age and gender, should be included in the models. The results reveal that age does not have a significant effect in any of the models, whereas gender does have significant main effects on both planning and execution in that males are faster than females. However, the effect of gender on the conditional dependency is not significant. Furthermore, the main conclusions (including the positive correlations between the random effects of planning and execution, the negative fixed dependency, and the comparison of the four models) remain the same after adding gender as a covariate. To simplify the presentation of the results and because the covariates do not affect the focal points of the results, we only present the results from the models without covariates.

### Results of Analysis 2

As in Analysis 1, the fixed dependency parameter *ω* (here it is the fixed effect of the residual planning time on the observed execution time) is estimated to be negative (see Table 4). Both the goodness-of-fit indices and the likelihood ratio test (see Table 5) suggest that all dependency models fit the data better than the ND model does and that the PSD and ISD models fit the data better than the GD model does. The likelihood ratio test results are significant even with *p*-values that are conservative due to boundary issues when the PSD and ISD models are involved. The results indicate that there is a negative conditional dependency of the execution time on the residual planning time and that the dependency varies across both persons and items. Note that, in Analysis 1, we have not found evidence for a variation of the dependency across persons when using the observed planning time as a predictor of the execution time. The difference between the two results may be related to the difference in the estimated standard deviation of the person-specific dependency, which is very small in Analysis 1 (see Table 2) and much larger in Analysis 2 (see Table 4).

**Table 4:** Dependencies of planning and execution (Analysis 2).

		GD model	PSD model	ISD model
Fixed Dependency	estimate	-0.09	-0.08	-0.08
	SE	0.02	0.02	0.04
	t value	-4.75	-3.46	-2.24
Random dependency	SD	-	0.15	0.10

**Table 5:** Goodness of fit of the ND, GD, PSD, and ISD models (Analysis 2).

Model	AIC	BIC	Log-likelihood	Likelihood ratio test	
				Compared to ND	Compared to GD
ND	1377.96	1398.29	-684.98	-	-
GD	1357.66	1383.07	-673.83	X^2^(1) = 22.30***	-
PSD	1343.39	1373.88	-665.70	X^2^(2) = 38.57***	X^2^(1) = 16.27***
ISD	1347.20	1377.69	-667.60	X^2^(2) = 34.76***	X^2^(1) = 12.46***

*p<0.05

**p<0.01

***p<0.001.

### Results of Analysis 3

A confirmatory factor analysis was first conducted with the correlated two-factor model without estimating conditional dependency. The results indicate that the model fails to fit the data well, *RMSEA* = 0.09, *CFI* = 0.86, *TLI* = 0.84. After adding the dependency per item (i.e., including residual correlations), the goodness of fit is clearly better, *RMSEA* = 0.07, *CFI* = 0.91, *TLI* = 0.89. The likelihood ratio test comparing the two models shows that the model with the dependencies fits the data significantly better: *X*^2^(10) = 68.34, *p* < 0.001. In the model with conditional dependency, the correlation between the latent variables of planning and execution is significantly positive, which is consistent with the hypothesis that planning and execution are positively correlated at the latent variable level. Moreover, among the estimates of item-wise residual correlations, four of the ten are significantly negative, three are negative but not significant, and three are positive and not significant. This is in line with the earlier findings of an overall negative dependency. The reason that not all item-wise dependencies are negative can be explained by the item-specific variation of the dependency found in the previous analyses (i.e., in the ISD model). Such item-specific variation indicates that the dependency varies across items, which could lead to non-negative dependencies for some items.

Finally, the correlations between the estimated residual correlations from the factor model and the estimated item-specific dependencies from the linear mixed models in Analyses 1 and 2 are found to be 0.64 and 0.95, respectively. The high correlations provide strong support for the robustness of the item-wise dependencies.

## Discussion

Considering the imbalance between the emphasis on problem solving and the lack of research on the relationship between the involved processes, this study focuses on two important components within problem solving: planning and execution. Evidence from the results supports the hypothesis that the relationship between planning and execution is complex and depends on the levels of the variables (i.e., the latent variable level and the manifest variable level).

At the latent variable level, a positive correlation between planning speed and execution speed has been found to be independent of the type of modeling, which provides robust evidence in support of the hypothesis of general mental speed [10]. As two typical cognitive processes, planning and execution may both rely on general mental speed and consequently demonstrate a positive correlation between them. Analogous with the finding that mental speed has a positive correlation with measures of intelligence [10,11,26], planning speed and execution speed are likely to be associated with problem-solving ability, which is considered to be involved in the game-based assessment of this study.

As for the relationship at the manifest variable level after controlling for latent variables, estimates of the fixed dependency parameter *ω* in the LMM analysis and estimates of the item-wise residual correlations in factor analysis are consistent with the negative dependency hypothesis. This suggests that it pays off for more efficient execution to spend more time on planning while the lack of planning results in a longer execution process, although the effect seems to depend on the item and to some extent on the person, as will be discussed further on. Unlike the positive correlation which represents the overall relation between planning and execution across persons and across items, this negative dependency describes the association between planning and execution per person-and-item pair after controlling for the latent variables. In this study, we have examined the conditional dependency in three different analyses. In the context of LMM, Analysis 1 tests the direct effect of the observed planning time on the observed execution time at the manifest variable level, to check whether more time spent on planning contributes to more efficient execution. Analysis 2 specifically focuses on the effect of the residual planning time on the observed execution time. The residual planning time is the extra time spent on planning (if the residual is positive) or the time spent less on planning (if the residual is negative) compared with the expected time based on the time intensity of the task and the planning speed of the respondent (i.e., the latent variable). Although both types of the conditional dependency (one type in Analysis 1 and the other in Analysis 2) are based on reasonable assumptions and have been inspected in previous studies [15,17], these two types have not yet been systematically discussed and compared in the literature. It is a topic of future studies to compare these two types of conditional dependency theoretically and in different kinds of applications. Analysis 3 explores the conditional dependency through the residual correlations in a factor analysis. The extremely high correlation (0.95) between the residual correlations in Analysis 3 and the item-specific dependencies in Analysis 2 is not surprising as they both rely on the residual planning time. Despite the differences among the three analyses, a negative dependency is always found at the manifest variable level independent of which of the three analyses is adopted. This negative dependency and the positive latent variable correlation have opposite signs and contain different information about the data.

Furthermore, the negative dependency has been found to vary to some extent across persons and more clearly across items. The person-specific dependency as found in Analysis 2 (but without clear evidence from Analysis 1) suggests that there are different types of problem solvers. Specifically, the benefit that execution takes from planning may be larger for some problem solvers than for others. This is perhaps because of differences in the planning quality. With the same residual planning time for a certain item, some people may be able to produce a better plan that helps execute an action more efficiently.

Analogously, the item-specific dependency shows that planning contributes more to execution for some items than it does for other items. A possible reason is that it is difficult to make plans at the very first for some items so that a longer planning time will not help much. Problem properties causing these differences should be explored in the future.

In addition, different from most latent variable model research, this study has placed much emphasis on the direct relationship between observed variables after controlling for latent variables. Without any doubt, it is reasonable to focus primarily on latent variables in some contexts, such as a context where only broad interindividual differences are of interest. However, when the relationship between two concepts is investigated in a more comprehensive and more detailed way, one should consider all types of associations between the concepts, including more direct effects between observed variables after controlling for latent variables. From this more comprehensive perspective, remaining dependencies between observed variables are no longer an imperfection of latent variable models, but a meaningful part of the total picture with important information that cannot be found at the latent variable level. As a result, conditional dependency should be given more attention in future latent variable model research, especially when parallel data are collected regarding the same items (e.g., response times and responses for the same items, and activations of two brain areas for the same cognitive activities).
